# Hyaluronan Hybrid Cooperative Complexes Injection as a Biostimulation for Postobese Skin Laxity in the Arm: A Histopathologic Study

**DOI:** 10.1093/asjof/ojad110

**Published:** 2023-12-08

**Authors:** Andrea Margara, Diala Haykal, Danilo Musella, Gilberto Bellia, Filippo Boriani

## Abstract

**Background:**

The Hybrid Cooperative Complexes of high and low molecular weight hyaluronic acids (HHCC) improve skin structure and bioactivity. Massive weight loss damages cellular composition and morphological structure of skin. An injective treatment of postobese skin consisting of HHCC may have a role in counteracting these histopathological alterations.

**Objectives:**

To analyze the histological effects of HHCC injection in the cutaneous tissues of massive weight loss patients suffering from arm laxity.

**Methods:**

Nine ex-obese patients with postweight-loss-related arm laxity and ptosis requiring brachioplasty were prospectively recruited at the first author's department. HHCC injection was performed on only 1 arm, which included 2 injective sessions separated by 30 days. One month posttreatment, patients underwent a bilateral brachioplasty, and the surgical specimens were histologically examined, searching for any variation in the cutaneous connective tissue following injections. Histology on treated specimens showed a statistically significant increased density of elastic fibers along with a lower fragmentation of the same fibers compared to the untreated tissue. Fibroblasts demonstrated a swollen appearance as if involved in a bioactivation process.

**Results:**

Treatment with HHCC increases the number of elastin fibers and determines a more regular elastin deposition and architecture, as well as the bioactivation of fibroblasts. The contralateral untreated area showed an irregular structure with elastosis and elastolysis.

**Conclusions:**

More studies are necessary, but histologically proven benefits are demonstrated in the HHCC-treated skins when compared with basal controlateral skin. These data support the use of HHCC formulations for the treatment of postobese skin laxity.

**Level of Evidence: 5:**

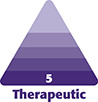

After bariatric procedures, macroscopic changes of skin and subcutaneous tissues are frequently massive and clinically well evident, but few studies have explored the underlying modifications in dermal and subcutaneous composition and architecture.^1-3^ Common alterations in postobese patients are diminished dermis vascularization, architectural collagen, and elastin fiber derangement, as in aging skin.

Modern aesthetic medicine treatments include a variety of products proposed to stimulate fibroblasts and more generally to counteract skin aging.^4^ Hyaluronic acid (HA) is one of these products and nowadays is currently used. However, HA rejuvenating/stimulating properties in massive weight loss patients’ skin have not been much investigated in the medical literature.

HA has physical, chemical, and rheological features that determine its high biocompatibility.^5-8^ The relevant density of HA, in the human dermis, allows its hydration, keeping at the same time, an adequate tissue volume which protects skin cells from mechanical damage. HA is a molecule with a critical involvement in skin aging.^7^ In fact, skin aging can be explained as an alteration targeting elastin, collagen, and ultimately the HA amount. The skin matrix is critical for mechanical elasticity, structural integrity, functional stability, and many other properties. Skin aging is also due to a number of tissue modifications in various skin layers, such as derangement of the epidermal–dermal border, reduction of the subepidermal elastin fiber network, and dermal atrophy.^9,10^

The loss of HA may determine pathophysiological alterations in fibroblasts and keratinocytes.^11,12^ The use of HA showed some benefits in terms of epidermal homeostasis barrier and tissue thickness in preclinical models.^13^ Since the 1980s, all these findings have been popularized and have led to the common use of HA injections for wrinkle improvement and rejuvenation treatments.^14^

The Hyaluronan Hybrid Cooperative Complexes (HCC) produced and distributed by IBSA Farmaceutici Italia Srl, Lodi, Italy, are a promising tool, recently introduced in regenerative and aesthetic medicine.^15,16^ Their bioeffect is an enhancement of skin structure and bioactivity, as they both promote the renewal of the stromal matrix and stimulate the cellular elements in terms of quantity and activity. A novel value of these complexes is their prolonged resistance to enzymatic digestion, even with no chemical cross-linking.

The interaction between fibroblasts and HCC formulation has been assessed in the recent past through in vitro biomodels.^13^ These have demonstrated the important biological response that increases collagen and elastin synthesis, thereby causing a global biorevitalization effect.

Massive weight loss determines relevant modifications in the cellular composition and morphological structure of skin tissue. These histologically evident changes include a decrease in the amount of collagen, particularly collagen III, and an alteration of the collagen network.^1-3^

An injective treatment of postobese cutaneous and subcutaneous tissue consisting of HCC may have a role in counteracting these histopathological alterations, by yielding increased production and deposition of collagen and elastin fibers in the dermal and subdermal layers. The aim of this study was to analyze the histological effects of HCC injection in the cutaneous tissues of massive weight loss patients who suffered from arm laxity, before an arm lift surgical procedure.

## METHODS

### Patients, Treatment, and Study Design

Nine consecutive postobese patients accessing our department between January and June 2022, with a postweight loss arm laxity and ptosis requiring brachioplasty, were considered for participation in the study and were included based on informed consent. The study was conducted in a private practice setting, so, accordingly to Italian laws, no Institutional Board review was necessary, but all patients consented to the study and to the anonymous use of the confidential information and the research was conducted according to the Declaration of Helsinki.

**Figure 1.**
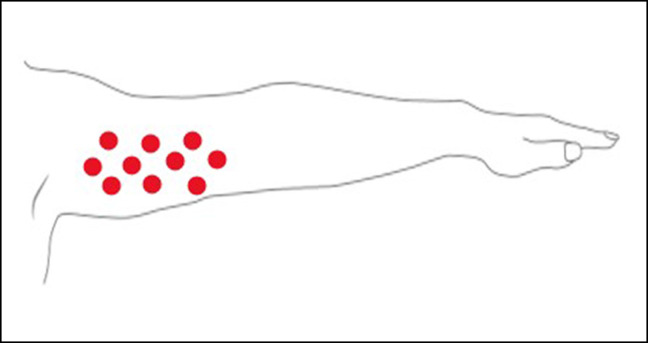
Schematic representation of the injection sites in the arm. This point could be positioned using a BAP Tool that helps in equally distancing each mark.

Included patients first underwent an incisional (punch) biopsy of 0.5 mm in diameter in the area of maximum laxity (most ptotic skin). This baseline biopsy was meant to explore the pretreatment histology of arm tissues after postmassive weight loss. Subsequently, an HCC injection protocol was performed in 1 of the 2 arms (Profhilo Body, IBSA Farmaceutuci Italia Srl), which involved 2 separate 30-day apart injection sessions; 10 boluses of 0.3 mL each spaced 2.5 cm apart were injected into the arm area shown in Figure 1 with the help of a template applied to the inside of the arms provided by the manufacturer of the hyaluronic acid-based HCC gel.

An important feature of the product injected is the ability to diffuse and the template provided by the manufacturer takes into account the diffusion area in determining the distribution of the holes to be used for injections mapping. As the injections were performed through the use of this template, the whole area covered by the template is to be considered as evenly perfused by the product. Therefore, the biopsies were not limited to the injection points but to the whole template-covered surface (Video).

HCC was injected in the area meant to be excised, into the dermis at the junction with the subcutaneous tissue. After each session, the patient was administered a cream containing a double-molecular-weight hyaluronic acid complex and a specific recipe consisting of a combination of 2 peptides and recommended to massage the area treated every day for 5 min. Thirty days after the injective treatment, patients underwent an upper limb dermolipectomy and the surgical specimens were sent for histology. Both the treated and the contralateral untreated arms were subject to histological examination. All the protocol has been performed within 2 months from the first injection, then after 30 days, patients have received the second injection treatment. All the treated areas of the patients’ arms were sampled 1 month after the second treatment when the patients were operated. Clinical follow-up of patients was not relevant to this study which is merely a histologic evaluation of surgical specimens.

### Histological Analysis

The basal biopsy (T0) was evaluated for its inflammatory status, for its cellular and stromal microenvironment. Specifically, density of the collagen matrix (in particular, the presence/absence of fibrosis processes); density of elastic fibers; presence and percentage of fragmented elastic fibers (elastolysis); density of solar/senile elastosiswere evaluated by Masson's trichrome staining and Van Gieson staining for elastic fibers. The specimen was stained with hematoxylin–eosin and with Masson's trichrome (Bio-optica), to stain the connective tissue, and collagen, while reticular and elastic fibers were identified with Van Gieson staining. In particular, these stainings clearly highlight the collagen fibers in blue and the elastic fibers in light red/pink.

The stromal cell populations (fibroblasts; macrophages and lymphocytes) were evaluated as the percentage of single cell populations/sqmm (semi-quantitative evaluation, ie, “by eye”); the presence of inflammatory processes and their arrangement; the presence of etiological agents related to inflammation (fungi; Demodex), through the following antibodies: CD68 (macrophages); Vimentin and CD34 (stroma); CD3 and CD20 (lymphocytes). PAS for fungi.

Quantitative count of the area occupied by elastic fibers as well as the number of intact and fragmented fibers was determined for each patient in the treated and untreated dermis and subcutaneous tissue. The ratio between the number of intact and fragmented elastic fibers was then calculated and taken as indicative of the regular architecture of elastic fibers.

### Statistical Analysis

Nonparametric *t*-test was used to determine the statistically significant difference between untreated and treated areas for these parameters.

## RESULTS

The mean age of the included patients was 43 (range, 35-50 years) and all of them were female. The mean preweight loss BMI was 47, and the mean postweight loss was 27. The only complications occurred while performing the injective treatment were superficial ecchymoses in 3 cases (33%). Dermo-subcutaneous tissue taken from 9 patients was examined, which included the area subject to treatment, clearly marked during surgery, and the adjacent and remote areas.

**Figure 2.**
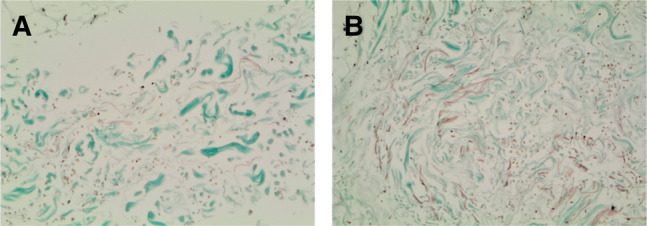
(A) Untreated cutaneous tissue: Masson staining: elastic fibers appear small, and fragmented within an interlobular septum. (B) Treated subcutaneous tissue: Masson staining: elastic fibers appear formed and linear within an interlobular septum: mildly increased density compared to untreated areas.

**Figure 3.**
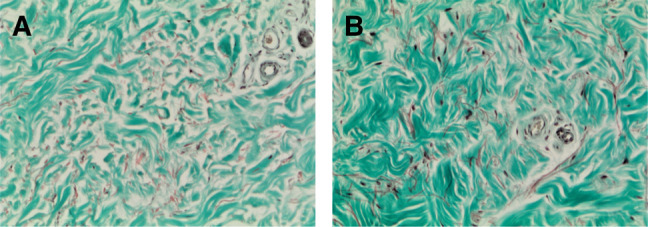
(A) Untreated cutaneous tissue. Different density of elastic fibers and increased volume of fibroblasts. Elastic fibers are small and fragmented, fibroblasts are small and rare. (B) Treated cutaneous tissue. Elastic fibers are formed and linear, fibroblasts are swollen and more numerous.

Although the number of patients studied was limited, in all of them, we could appreciate differences both in the dermal and in the subcutaneous layer. In the subcutaneous layer, in the injected areas, an increase in the density of the elastic fibers together with a lower fragmentation of the same fibers is noticeable compared to untreated tissue (Figures 2, 3A, B). From the images taken, it was possible to quantitatively determine some parameters such as the percentage of histological area occupied by elastic fibers (%FE), the number of fragmented or intact elastic fibers, and the ratio between intact and fragmented fibers, which represents an index of regular architecture.

**Figure 4.**
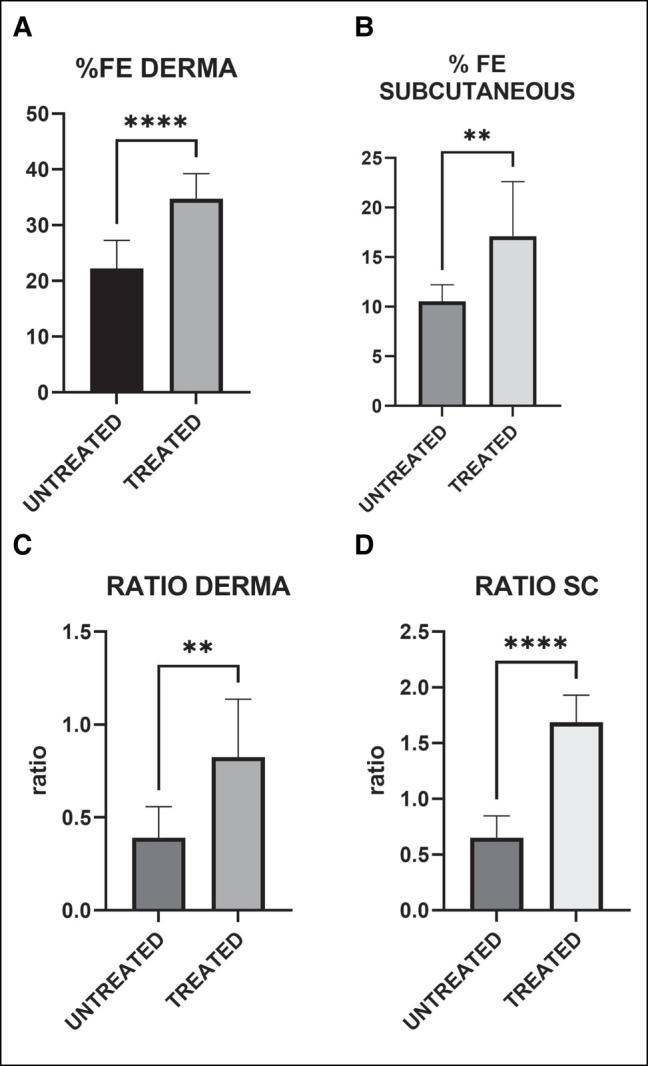
Quantitative analysis of %FE (A, B) and ratio between intact and fragmented elastic fibers (C, D) in dermis (A, C) and subcutaneous tissue (B, D). The values are the mean and SD for the 9 patients. **P < .01, ****P < .001 (*t*-test). FE, elastic fiber; SD, standard deviation.

**Figure 5.**
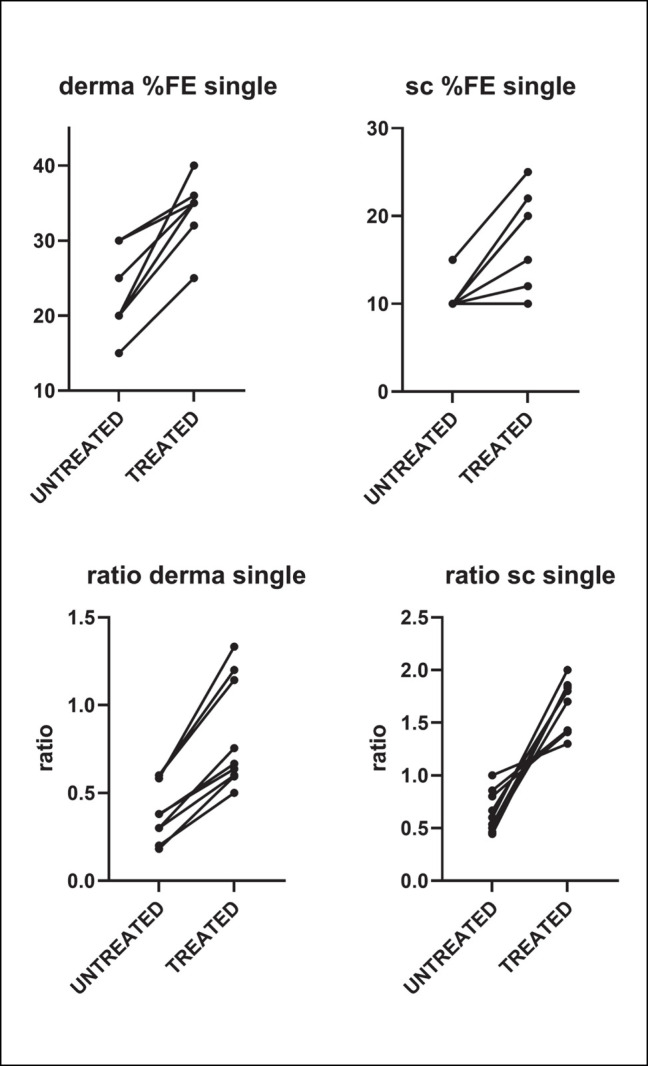
Changes between untreated and treated areas in the total area occupied by fibers (upper panels) and in the ratio between intact and fragmented elastic fibers (lower panels) in the 9 single patients. Each point represents a patient. FE, elastic fibers.

Figure 4 reports the results of the analysis conducted in dermis (Panels A and C) and subcutaneous tissue (Panels B and D). Panels A and B report the %FE, while Panels C and D report the ratio between fragmented and intact elastic fibers. As it can be seen from the graphs, in all the areas and for all the parameters analyzed, the treatment was able not only to enlarge the area occupied by fibers, but also to increase the ratio between intact and fragmented elastic fibers. Importantly, these improvements were statistically significant. Furthermore, a clear amelioration was observable for all the 9 patients, as shown in Figure 5.

## DISCUSSION

The results of this study showed an increased number of elastin fibers and a more regular deposition of elastic fiber architecture in the HCC-treated specimens. The contralateral untreated area showed an irregular structure with elastosis and elastolysis. Moreover, fibroblasts appear to be bioactivated by HCC treatment, which means that in a longer follow-up, we could expect a more abundant deposition of collagen fibers and an increase in the collagen network. These results are in line with previously published in vitro studies, demonstrating the same effects in cell cultures of keratinocytes and fibroblasts, with an increased transcript of elastin and collagen as shown by real-time PCR.^16^

Another confirmation of the current study is the ultrasound and patient- and physician-reported outcome research by Laurino et al,^15^ in which an improvement of skin tone, elasticity, hydration, and turgor was visible after HCC-based treatment. This is a preliminary study and, despite the limited number of included cases, the results are promising with regard to the important biological response that the HCC formulation achieved on the elastin and collagen fiber synthesis in damaged skin, possibly suggesting an effective way to perform antiaging biostimulating treatments. The beneficial effect of the treatment was observable in all the 9 patients, and the quantitative results were highly statistically significant, thus reinforcing the efficacy of HCC formulation. A longer term histological follow-up is not easily achievable, because it would entail further punch biopsies in patients whose surgical pathway is already long and multistep, in a body area where their perception is to have achieved a definitive result. However, subsets of motivated patients for this research might be found for future studies.

## CONCLUSION

This is the first study investigating the effects of HCC injections in the massive weight-loss arm. The same deformity was treated with regular HA by Distante et al,^17^ who demonstrated similar results; however, their evaluation was based on biophysical parameters rather than histology.

The current protocol was not designed to separate the effects of morbid obesity vs those of rapid weight loss, because biopsies in the weight-gain phase and during bariatric intervention were not included in the methods. This limitation can be addressed in future studies, as there are reasons to believe that both huge BMI elevation and reversal of fat body mass after successful bariatric intervention alter subcutaneous collagen scaffold.

The results showed for the first time a statistically significant increased number of elastin fibers and a more regular deposition of elastic fibers architecture as well as a bioactivation of fibroblasts in the HCC-treated skins when compared with basal controlateral skin. These data support the use of HCC formulation for the treatment of obese skin laxity.

## Supplementary Material

ojad110_Supplementary_Data
